# Neighbourhood characteristics as a predictor of adherence to dietary recommendations: A population-based cohort study of Finnish adults

**DOI:** 10.1177/1403494820971497

**Published:** 2020-11-25

**Authors:** Hanna Lagström, Jaana I. Halonen, Sakari Suominen, Jaana Pentti, Sari Stenholm, Mika Kivimäki, Jussi Vahtera

**Affiliations:** 1 Department of Public Health, University of Turku and Turku University Hospital, Finland; 2 Centre for Population Health Research, University of Turku and Turku University Hospital, Finland; 3 Health Security, Finnish Institute for Health and Welfare, Finland; 4 School of Health Sciences, University of Skövde, Sweden; 5 Clinicum, University of Helsinki, Finland; 6 Department of Epidemiology and Public Health, University College London, UK

**Keywords:** Neighbourhood socioeconomic disadvantage, population density, dietary recommendations, dietary habits

## Abstract

*Aims:* To investigate the association of six-year cumulative level of socioeconomic neighbourhood disadvantage and population density with subsequent adherence to dietary recommendations, controlling for preceding dietary adherence, in adults in Finland. *Methods:* Population-based Health and Social Support (HeSSup) study participants from four age groups (20–24, 30–34, 40–44 and 50–54 years at baseline in 1998). Data on diet and alcohol consumption were obtained from the 2003 and 2012 surveys and information on neighbourhoods from Statistics Finland Grid database (*n* = 10,414 men and women). Participants diet was measured as adherence to Nordic Nutrition recommendation (score range 0–100). *Neighbourhood disadvantage* was measured by median household income, proportion of those with primary education only and unemployment rate, and *population density* by the number of adult population between years 2007 and 2012. Linear models were used to assess the associations of neighbourhood characteristics with the score for adherence to dietary recommendations in 2012. *Results:* Cumulative neighbourhood socioeconomic disadvantage was associated with slightly weaker (1.49 (95% confidence interval (CI) −1.89 to −1.09) point decrease in dietary score) adherence while higher population density was associated with better (0.70 (95% CI 0.38−1.01) point increase in dietary score) adherence to dietary recommendations. These associations remained after controlling for prior dietary habits, sociodemographic, chronic cardio-metabolic diseases, and severe life events. ***Conclusions*: These longitudinal findings support the hypothesis that neighbourhood characteristics affect dietary habits.**

## Introduction

Poor diet is among the leading modifiable risk factors of premature mortality and morbidity [1–3]. In addition to individuals’ socioeconomic status [4], social [5] and physical [6] characteristics of residential neighbourhoods might be linked to differences in dietary habits and food consumption. Observational studies suggest that healthy dietary habits are more common among residents living in affluent neighbourhood than in disadvantaged neighbourhoods [7–10], but that may also depend on population density as dietary habits have been found to vary between sparsely populated areas and more densely populated urban areas [11]. Limitation of the earlier evidence is that the findings are mainly based on cross-sectional data or single food items, which may partly explain the mixed findings [9–13].

We have already shown cross-sectionally, that people living in the highest versus lowest socioeconomic status neighbourhoods had better adherence to dietary recommendations [8]. In the present study, we examined the longitudinal associations of cumulative socioeconomic disadvantage and population density in residential neighbourhoods with adherence to dietary recommendations adjusting for prior dietary habits.

## Methods

### Study population

Health and Social Support (HeSSup) is a follow-up study commenced in 1998 (*n* = 25,901) representative of the Finnish Population in four age groups (20–24, 30–34, 40–44 and 50–54 years at baseline) [14]. We included participants with information on diet and alcohol consumption at the first follow-up survey, that is baseline of this study (2003, *n* = 19,629; response rate 76%) and at the next follow-up survey (2012, *n* = 13,050; response rate 50%), and excluded those who had missing information on home addresses (*n* = 17), on neighbourhood socioeconomic status because of living in sparsely populated areas (*n* = 1582), had not responded to the minimum of five food item questions (*n* = 27) or had missing dietary score at baseline (*n* = 1010). The final study population for this study was 10,414.

### Neighbourhood characteristics

Data on neighbourhood factors were obtained from the Statistics Finland’s grid database for the year 2009. This database contains information based on all Finnish residents on social and economic characteristics at the level of 250 × 250 m^2^ map squares [15]. For each participant we obtained geocoded residential addresses and dates of moves for the years 2007 to 2012 from the Population Register Center of Finland; these data were positioned to the Statistics Finland Grid Database to calculate residential time-weighted cumulative mean exposure to the neighbourhood characteristics during this six-year residential exposure window. *Neighbourhood disadvantage* was measured for those aged > 18 years using median household income (coded as additive inverse), proportion of those with primary education only, and unemployment rate from each map square. For each of the three features, we derived a standardized *z* score (mean = 0, SD = 1) and the disadvantage scores were then calculated by taking the mean value across the three *z* scores, higher score indicating higher disadvantage. *Population density* was measured as the number of population > 18 years within each map square from the Statistics Finland database [15] and the mean number was standardized for analyses.

### Dietary habits

The participants reported their habitual frequency of eating or drinking on selected dietary components in 2003 and 2012. From the short non-validated food frequency questionnaire, 10 items or groups (dark bread (⩾ 2/day), pastries and sweets (⩽ 1–2/week), fat free milk (⩾ 1/day), sausages (⩽ 1–2/week), red meat (⩽ 1–2/week), chicken or turkey (⩽ 1–2/week), fish (⩾ 1–2/week), fresh fruits and berries (⩾ 2/day), vegetables (⩾ 2/day) and alcohol use (< 10 g women/day, 20 g men/day) were used to form a dietary index to describe the adherence to dietary recommendations, which are in line with Nordic Nutrition Recommendation 2004 [16]. Each recommended choice provided one point for the index, so the overall score varied from 0 to 10, the maximum indicating perfect adherence to recommendations [8]. For the analyses, we multiplied the score by 10 to have a percentage scale ranging from 0 to 100.

### Covariates

Information on covariates was from the 2012 survey. Sociodemographic factors included age, sex, marital status and education. Education was categorized into four levels: (a) basic education, (b) high school/vocational education, (c) college and (d) university or higher education. Marital status was categorized as living alone versus married/cohabiting. Chronic cardio-metabolic diseases (hypertension, diabetes, atrial fibrillation, ischemic heart disease and/or cerebrovascular disease) (no/yes) were measured from a check list of doctor diagnosed diseases. Severe financial difficulties (no/yes), divorce and death of spouse within the past five years (no/yes) were measured from a list of recent life-events.

### Statistical analyses

We used generalized linear models (genmod procedure in SAS) for assessing the associations of cumulative neighbourhood disadvantage and population density from 2007 to 2012 with the score for adherence to dietary recommendations in 2012. We assessed the associations using unadjusted and 2 adjusted models (for dietary score from year 2003, and additionally for sex, age, marital status, education, chronic cardio-metabolic diseases, severe financial difficulties, death of spouse and divorce). We first examined the associations of neighbourhood disadvantage and population density with diet separately, and then, to examine their independent effects, as mutually adjusted.

## Results

Baseline characteristics are shown in Table I. In both surveys 2003 and 2012 about half of the 10 food groups were consumed as recommended, as indicated by a mean dietary score of 51.3 (SD 16.8) and 54.3 (SD 16.5), respectively (Table I).

**Table I.** Descriptive statistics of the study population (*n* = 10,414)^a^ neighbourhood variables and dietary score from year 2003 and 2012.

Variable		*n*	%	Mean	SD
**Sex**	Men	3853	37		
	Women	6561	63		
**Age**				52.8	11.4
	34–38	2254	22		
	44–48	2130	20		
	54–58	2729	26		
	64–68	3301	32		
**Marital status**	Single	2593	25		
	Cohabiting	7777	75		
**Level of education**	Basic	950	9		
	High school^b^	2739	26		
	College	3049	29		
	University	3609	35		
**Cardio-metabolic diseases**	No	7794	75		
	Yes	2593	25		
**Financial difficulties** ^c^	No	8735	84		
	Yes	1631	16		
**Death of a spouse** ^c^	No	10184	98		
	Yes	182	2		
**Divorce** ^c^	No	9583	92		
	Yes	783	8		
**Diet Score** ^d^	Year 2012	10414		54.3	16.5
	Year 2003	10414		51.3	16.8
**Residential neighbourhood** ^e^	Density^f^	10414		180.3	247.1
	Disadvantage	10414		−0.17	0.79

a Health and Social Support (HeSSup) a follow-up survey 2012.

b Including vocational school.

c Over the last five years.

d Mean score for adherence to Nordic Nutrition Recommendation 2004; total points based on 10 individual food items/groups for the dietary index scaled so that score can range from 0 to 100.

e Participant’s residential 250 × 250 m^2^ square and the eight surrounding 250 × 250 m^2^ squares.

f Adult population density within the 250 × 250 m^2^ neighbourhood.

Studying separately, in the unadjusted model, one SD increase in neighbourhood socioeconomic disadvantage was associated with 1.49-point decrease in the dietary score (95% CI −1.89 to −1.09), whereas one SD increase in neighbourhood population density was associated with 0.70-point increase in dietary score (95% CL 0.38–1.01) (Table II). Adjustment for year 2003 dietary score roughly halved the effect estimates, and only association for socioeconomic disadvantage and dietary score remained after further adjustments for sociodemographic factors, morbidity and life events.

**Table II.** Dietary score (95% confidence intervals (CI)) by one SD increase in population density and one SD increase in neighbourhood disadvantage.

Exposure	Model 1			Model 2			Model 3		
	Estimate	95% CI		Estimate	95% CI		Estimate	95% CI	
Separately									
**Disadvantage**	**−1.49**	−1.89	−1.09	**−0.90**	−1.25	−0.55	**−0.67**	−1.04	−0.30
**Population density**	**0.70**	0.38	1.09	**0.31**	0.04	0.59	0.22	−0.06	0.51
In the same model									
**Disadvantage**	**−1.54**	−1.94	−1.14	**−0.93**	−1.28	−0.58	**−0.68**	−1.05	−0.31
**Population density**	**0.77**	0.45	1.08	**0.36**	0.08	0.64	0.24	−0.04	0.52

Model 1: unadjusted.

Model 2: adjusted for dietary score from year 2003.

Model 3: adjusted for dietary score from year 2003, sex, age, marital status, self-reported education, chronic cardio-metabolic diseases, severe financial difficulties, death of spouse and divorce.

When mutually analysing neighbourhood socioeconomic disadvantage and population density, one SD increase in socioeconomic disadvantage was associated with 1.54-point decrease in the dietary score (95% CL −1.94 to −1.14), whereas one SD increase in neighbourhood population density was associated with 0.77-point increase in dietary score (95% CL 0.45–1.08) in the otherwise unadjusted model (Table II). When controlling for baseline dietary score, both disadvantage and density remained independently associated with dietary score. After adjusting for all covariates, only socioeconomic disadvantage remained significantly associated with the dietary score.

## Discussion

We observed that higher cumulative neighbourhood socioeconomic disadvantage was associated with worse adherence while higher population density was associated with better adherence to dietary recommendations. These associations remained after controlling for prior dietary habits and association for disadvantage also remained after further adjustments for a wide range of individual-level risk factors.

This is, to the best of our knowledge, one of the first studies to examine associations of cumulative exposure to socioeconomic and urban neighbourhood characteristics with adherence to dietary recommendations using a longitudinal setting where prior dietary habits have been taken into account. Our findings on neighbourhood population density, representing built urban environment, suggest that densely built environments offer better opportunities for gaining and maintaining healthy diet [6,10,17,18] by offering better transportation systems and food availability [17,18]. However, as the adjustments attenuated this finding, it is likely that low population density alone does not explain poor dietary choices in the Finnish, or Nordic, context. Associations between socioeconomic disadvantage and diet, on the other hand, may be explained by the poorer access to healthy foods because of financial resources, lack of neighbourhood shops, or worse selection of healthy foods in shops of the residential area [6,13].

The strengths of our study are its prospective design, objective measurement of cumulative neighbourhood socioeconomic disadvantage in combination with population density, and repeated measurements of dietary habits. We also controlled for important time-dependent covariates, such as major chronic illnesses, severe financial difficulties, divorce and death of a spouse, which are potentially associated with change in the area of residence and dietary habits.

However, some limitations are also acknowledged. The study population was female-dominated and middle-aged (age range 34–69 years), which limits the generalizability of the findings to other population groups. Use of self-reported dietary data may have resulted in bias, as respondents may have systematically under- or over-reported the consumption of individual food items. Nevertheless, trait-like individual differences are an unlikely source of major bias as we were able to control for preceding dietary index. Furthermore, our dietary index is based on a short version of a food frequency questionnaire, and although we were able to include all those food groups for which the justification for the recommendation was obtained [16], food frequency questionnaires are less precise than those based on weighted records. However, our measure covers a range of specific foods and is feasible for large-scale cohort studies [19], such as ours.

The result of this study showed that high neighbourhood socioeconomic disadvantage and low population density were associated with worse adherence to dietary recommendations. Therefore, public health efforts to improve dietary habits may benefit from identification of several living environmental factors simultaneously.
